# Potential Use of Kaolin in Viticulture: Physiological Basis and Future Perspectives

**DOI:** 10.3390/plants15081276

**Published:** 2026-04-21

**Authors:** Leonor Deis, Juan Martínez-Barberá, Francesca Fort, Pedro Balda, Alicia Pou, Andrea Mariela Quiroga, Raúl Ferrer-Gallego

**Affiliations:** 1 Fisiología Vegetal, Universidad Nacional de Cuyo, Mendoza M5500, Argentina; 2 Oenological Technology Group (TECNENOL), Department of Biochemistry and Biotechnology, Facultad de Enología, Universidat Rovira i Virgili, Sescelades Campus, C/Marcel·lí Domingo, 1, 43007 Tarragona, Spain; mariafrancesca.fort@urv.cat; 3 PhD Program in Food Science, Technology and Management, Polytechnic University of Valencia (UPV), Camí de Vera, s/n, Algirós, 46022 Valencia, Spain; juan@ingroin.com; 4 Department of Ecology, Desertification Research Centre, CIDE (CSIC-UV-GV), 46113 Moncada, Spain; raul.ferrer@csic.es; 5 Department of Food and Agriculture, Scientific and Technology Complex, Universidad de La Rioja, c/Madre de Dios, 51, 26006 Logroño, Spain; pedro-jose.balda@unirioja.es; 6 Instituto de Ciencias de la Vid y del Vino—ICVV (CSIC, UR, GR) Finca La Grajera, 26007 Logroño, Spain; alicia.pou@icvv.es; 7 FICA, Universidad Nacional de San Luis, Ruta Provincial N° 55 Extremo Norte, Villa Mercedes D5730EKQ, San Luis, Argentina; amquir@yahoo.com.ar

**Keywords:** anthocyanins, climate change, *Empoasca vitis*, grapevines

## Abstract

Since ancient times, clays have been used to protect plants from insects and excessive sunlight. Today, their potential use is being re-evaluated as a tool to mitigate the effects of climate change and to manage emerging pests. This review synthesizes and compares findings from studies conducted in different regions of the world. Kaolin forms a reflective film on leaves and fruits, lowering tissue temperature. In warm climates, this temperature reduction can contribute to improved physiological parameters including net assimilation and water use efficiency; however, these responses are strongly influenced by additional factors. It may also affect some oenological characteristics of grapes (acidity, pH, and phenol content, particularly anthocyanins), thereby improving the overall chemical composition of grapes and wines, particularly in terms of acidity, pH and phenolic content. In addition, kaolin has been shown to reduce damage caused by the grape leafhopper (*Empoasca vitis*, *Jacobiasca lybica*, among others) to levels comparable to those achieved with synthetic pesticides. However, responses vary depending on different factors, such as application timing, dose, cultivar and climate. Overall, kaolin represents a sustainable strategy for mitigating climate change effects on fruit quality and for supporting ecological pest management.

## 1. Introduction

Historical records from Egypt, Mesopotamia+, and China indicate that clays have been used in agriculture since at least 2500 BC for crop protection and soil improvement. The term kaolin, of Chinese origin, refers to a material that has historically been used in ceramic formulations [1] and in coating the trunks of fruit trees. This practice served to protect the plants from insects and excessive sunlight [2]. In Greece, evidence from around 300 BC indicates that kaolin was also used for drying grains and fruits due to its water-absorbing properties [3].

Kaolin is a natural clay mineral, also known as kaolinite, that belongs to the phyllosilicate group. It is an aluminum silicate (Al_2_Si_2_O_5_(OH)_4_) composed of layered aggregates held together by hydrogen bonds, which confer distinctive properties compared to other clays, including its non-expandable nature [4]. Kaolin is characterized by high reflectance, low nutrient retention, and chemical inertness [5]. It forms through chemical weathering of silicate minerals under specific climatic and drainage conditions. Major deposits occur in the United States, China, and Brazil [6].

**Figure 1:**
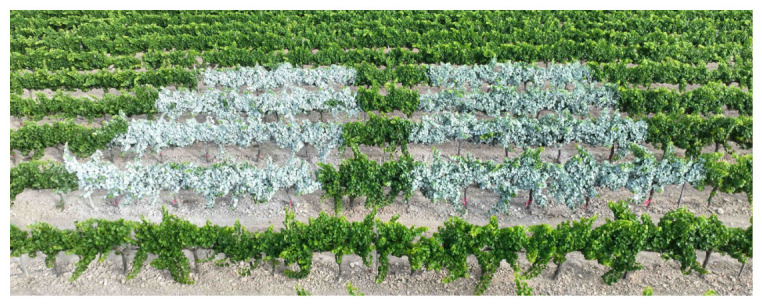
Kaolin application in the experimental vineyard of Marselan grape variety, sprayed with a backpack sprayer at constant pressure of 10 bars at two concentrations: 10% *w*/*v* (**left**) and 5% *w*/*v* (**right**).

Agricultural formulations are produced by grinding, sieving, bleaching, and calcining the extracted rock [6], the resulting formulation (wettable powder) or aqueous suspensions. Its earliest documented agricultural use involved the whitewashing tree trunks to reflect sunlight and prevent scorching in China [1]. It is currently marketed as a micronized formulation for foliar application, where it forms a protective film. Kaolin applications are now referred to as Kaolin Particle Film (KPF) [6,7]. As shown in Figure 1, kaolin application results in a whitish canopy, with the intensity of the white coating increasing with the applied dose. Due to the chemical and physical characteristics of kaolin, it reflects incident light, thereby reducing the temperature of the underlying surface [7,8]. The wavelength most strongly reflected correspond to ultraviolet and infrared radiation [9].

This review synthesizes and compares published results of the use of kaolin in viticulture to mitigate the effects of climate change.

## 2. Effects of Kaolin on Foliar Temperature, Energy Balance and Photosynthesis

**Figure 2:**
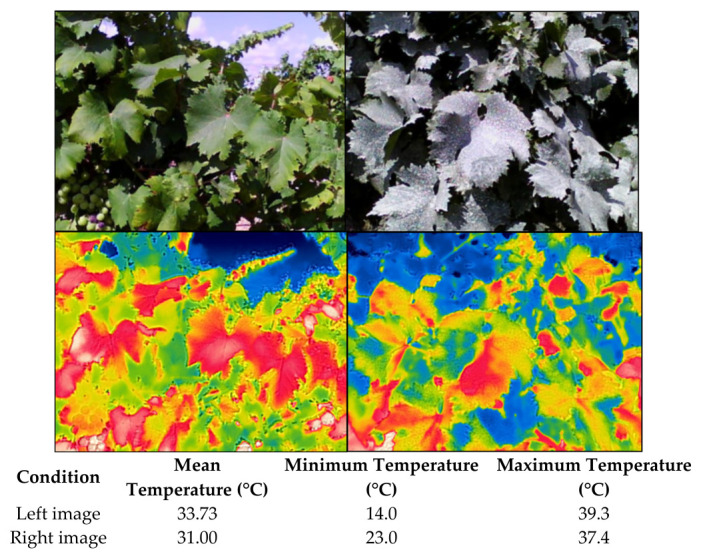
Untreated 33.73 °C, range 14/39.3 °C (**left**), and kaolin-treated 31 °C, rage 23–37.4 °C (**right**), Moscatel vines in Valencia, with the corresponding thermal images shown below. In thermal images, blue indicates cooler areas, green represents intermediate temperatures, and red corresponds to warmer zones.

When kaolin is applied to cultivated plants, the resulting film reflects incident solar radiation, thereby reducing the amount of energy absorbed by the plant surface and altering the overall energy balance. Several studies have demonstrated that kaolin-coated surfaces experience substantial temperature reductions. In grapevines, foliar temperature has been reported to decrease by approximately 2.8 °C, and berry temperature by around 2 °C in specific studies [8,10], although the magnitude of these effects varies depending on environmental conditions and the experimental context. In Sauvignon Blanc grown under temperate and humid climate conditions [10,11,12] berries without kaolin applications showed temperatures up to 5.7 °C higher than those sprayed with kaolin [10,13]. In contrast, in Cabernet Sauvignon cultivated in a Mediterranean climate, the temperature reduction was only 1 °C [10,14]. In Syrah vineyards located in hot and arid regions, decreases of 2.3 °C were observed during the warmest vintages [15]. In Türkiye, the Sultan 7 cultivar showed a decrease of 2.84 °C on the western canopy and 2.29 °C on the eastern side [8]. Pinot Noir in Italy exhibited a reduction of up to 6 °C [16]. These effects were smaller in Sauvignon Blanc under adequate water availability (0.3 °C) whereas under water stress the thermal difference increased to 1 °C [16]. In Monastrell grown under very warm conditions in Jumilla (Murcia, Spain), leaf temperature decreased by1.3 °C [17]. Temperature differences close to 3 °C were also observed in other Mediterranean areas, such as in Valencia (Spain), for the Moscatel variety (unpublished data). Figure 2 illustrates these differences, with untreated vines reaching 33.75 °C and kaolin-treated vines (10%) averaging approximately 31 °C.

Overall, the magnitude of the cooling effect of kaolin varies with climate, solar radiation, plant water status, and cultivar-specific traits [18].

In other crops, such as apples, kaolin can lower skin temperature by approximately 6–8 °C and leaf temperature by approximately 3–5 °C [6,15]. These larger reductions likely reflect differences in fruit size, exposure to sunlight, and epidermal characteristics. Apples have been reported to show greater temperature reductions; however, this response is influenced by multiple factors, including canopy structure, fruit exposure, transpiration, and epidermal traits. Regarding leaves, temperature differences between species are largely due to the differences in stomatal density. Grapevine leaves with higher stomatal density dissipate heat more efficiently, making the relative cooling effect of kaolin less pronounced than in apples.

Nevertheless, lower leaf temperature can reduce thermal stress, thereby limiting potential damage to cellular membranes, photosystem II, and Rubisco activity [19,20]. This may contribute to improved photosynthetic performance and can be associated with reduced water loss via transpiration, as the vapor pressure gradient between stomata and the surrounding air is lowered [21]. High temperatures can lead to overexcitation of the photosystems, generating reactive oxygen species that induce lipid peroxidation [22]. Consequently, lower temperatures enhance the efficiency of photosystem II [23] and enzymatic activity, thereby increasing CO_2_ fixation [24]. Another response is an increase in stomatal conductance [25], which has been reported in several studies where kaolin reduced leaf temperature and photoinhibition, allowing stomata to remain partially open under water deficit while maintaining relatively higher photosynthetic rates [26]. This response may contribute to improved water use efficiency (WUE); however, WUE depends on the balance between carbon assimilation and transpiration, and partial stomatal opening may also increase water loss. In grapevines, increases in WUE have been reported under different crop conditions and across varieties [27,28]. For example, in Merlot and Viognier, WUE improved under water deficit conditions, whereas no substantial improvements were observed under irrigation regimes [29]. In contrast, in Cabernet Sauvignon, seasonal WUE improved regardless of irrigation [29]. Under water deficit, stomatal closure limits water loss. If kaolin lowers leaf temperatures, stomata may remain partially open, allowing CO_2_ assimilation, thereby increasing WUE. Conversely, when plants are irrigated, stomata are already open and transpiration is not limiting; therefore, kaolin does not substantially modify WUE.

At the metabolic level, kaolin application enhances photosynthetic performance through two distinct mechanisms. First, the reduction in leaf temperature enhances Rubisco activity, resulting in increased carbon fixation [10,30]. Second, by decreasing incident light intensity, it improves photosystem II efficiency (up to 67% in Touriga Nacional), reducing the production of reactive oxygen species and further enhancing carbon assimilation [10,30]. The electron transport rate increased by up to 73%, even up to six weeks after kaolin application [14,30]. The enhancement in photosystem II efficiency and electron transport rate indicates a reduction in photoinhibition, particularly in warm and dry conditions [23]. However, these improvements in photosynthetic performance do not necessarily translate into increased yield, which may be constrained by sink limitations or carbon allocation patterns within the plant. Furthermore, kaolin increased the concentrations of total chlorophyll and carotenoids, which play a crucial role in protecting against photoinhibition [10,31]. At the same time, reducing leaf temperature also decreases respiration rates, thereby increasing net assimilation [32]. Although these physiological effects were evident, the increase in CO_2_ assimilation was not reflected in pruning weight or yield [25,30], possibly due to sink limitations or constraints in carbon allocation within the plant. While total yields were not affected, an effect on berry weight in Merlot was found [25].

These physiological responses are particularly relevant in the context of climate change, where the interaction between water scarcity and rising temperatures constrains vine performance. Higher temperatures accelerate phenology, causing earlier budding and berry ripening. This earlier accumulation of sugars induces a decoupling between technological ripeness and polyphenol ripeness [33]. This mismatch negatively affects wine quality, altering sugar and phenolic accumulation as well as acidity and pH at harvest [16,34].

Furthermore, kaolin has been shown to influence hormonal regulation, particularly abscisic acid (ABA) and indole-3-acetic acid (IAA) dynamics under stress conditions [28]. More recent studies suggest that these effects may be enhanced when kaolin is combined with other compounds, such as silicon, indicating a potential synergistic effect on plant physiological responses [35,36].

As light is reflected by kaolin-coated tissues, tissue temperature decreases and photosynthesis improves, as previously described. Furthermore, plant water status is improved under drought conditions [24,37]. Thus, in contrast to the negative effects resulting from the interactions between water and thermal factors that can limit net assimilation and crop quality, kaolin represents a sustainable cultivation strategy that warrants further evaluation.

Studies conducted to date have reported substantial variability in the magnitude and direction of kaolin’s effects on leaf temperature and photosynthesis. This variability likely reflects differences in microclimate conditions, plant water status, cultivar specific genetic traits and even intrinsic heat stress tolerance mechanisms. Even so, kaolin shows strong potential as a climate change mitigation tool in Mediterranean vineyards. Its effectiveness, however, must be evaluated on a case-by-case basis.

## 3. Impact on Secondary Metabolism and Oenological Quality

Based on the advantages of kaolin for energy balance, the effects on grape quality have been evaluated. Table 1 summarizes the cultivars studied, the climatic conditions of each trial, the kaolin dose applied, and the main physiological and oenological responses observed.

Regarding technological maturity, evidence indicates that kaolin did not induce differences in the accumulation of soluble solids in Touriga Nacional and Touriga Franca [32]. In some studies, kaolin has been associated with lower °Brix and potential alcohol; however, these effects are not consistent across cultivars and environmental conditions. In contrast, in Viognier, soluble solid accumulation increased by up to 11% [25]. Beyond sugars accumulation, kaolin has been shown to modify pH, total acidity, anthocyanin concentration, and total polyphenol index (TPI) in berries, and these effects being reflected in the resulting wines. In general, kaolin tends to reduce the potential alcohol in wines [31,36], consistent with an approximate 8.9% decrease in °Brix. However, in red varieties such as Mara, Touriga, and Merlot, no differences in alcohol concentration have been reported [25,38,39]. Lower berry temperatures under kaolin treatments are associated with increased total acidity (+8%), and reduced pH (4%) [32]. Among organic acids, the concentration of malic acid increased by 11.1% and tartaric acid by 7% [32]. These changes in berry acidity were subsequently reflected in the wines.

Musts from grapes treated with kaolin showed lower pH values [30] due to a decrease in respiratory degradation at high temperatures [32]. These effects highlight kaolin’s potential as a tool to counteract the acid loss typically associated with climate warming. Another aspect evaluated was the response of polyphenolic compounds (Table 1). Reducing berry temperature has been associated with the stimulation of the biosynthetic pathway of these compounds, increasing total polyphenol content by 16% [26] and enhancing the anthocyanin concentration [31,40]; however, these responses cannot be attributed solely to temperature reduction, as other factors such as light exposure, sugar accumulation, and hormonal regulation may also play important roles.

In red wines, variable results have been reported. In vineyards under no water deficit conditions, anthocyanin content increased by up to 19% following kaolin application [39,41]. In Touriga Nacional and Franca, an increase in anthocyanins was observed during ripening, but these differences were not always detected in the final musts [32]. The increase occurred mainly in the glycosylated fraction, and in Touriga Franca, higher levels of delphinidin-3-O-coumarylglucoside were observed [32]. Although delphinidin is not a major anthocyanin in the berry, its acylation provides greater color stability in wine. A similar increase was detected in the concentration of flavanols [23].

**Table 1:** Effects of kaolin application on vine physiology and grape and wine traits under different cultivars and climatic conditions.

Cultivar	Climate	Dose (% *w*/*v*; Sprays)	Application Timing	Δ Leaf/ Berry T°	Leaf Physiological Response	Berry/Wine Response	References
Sauvignon blanc	Temperate–humid (Uruguay)	2–3; 1–2 sprays	Pre-veraison	↓↓↓ (−5.7 °C)	↑ Photosynthesis	↓ Alcohol; ↑ aroma balance	Coniberti et al., 2013 [13]
Cabernet Sauvignon	Mediterranean (Chile)	3; repeated	Multiple	↓ (−1 °C)	↑ WUE	=Phenolics; = yield	Lobos et al., 2015 [14]
Cabernet Sauvignon	Arid (USA)	6; not specified	Not specified	↓	↓ Net assimilation	↑ Anthocyanins; ↑ °Brix	Shellie & King, 2013 [40]
Verdejo	Continental (Spain)	5; 3 sprays	Pre-veraison	↓	↑ PSII efficiency	↓ pH; ↑ colour (L*)	Azuara et al., 2023 [30]
Malbec	Arid (USA)	6; not specified	Not specified	=	↓ WUE; ↓ assimilation	↑ Anthocyanins; ↑ phenols	Shellie & King, 2013; Shellie & Glenn, 2008 [25,40]
Pinot noir	Continental (Italy)	3; 2 sprays	Pre-veraison	↓↓↓ (−6 °C)	↑ PSII efficiency	↑ Colour; delayed ripening	Frioni et al., 2019 [16]
Syrah	Hot–arid (Portugal)	2–4; 2–4 sprays	Multiple	↓↓ (−2.3 °C)	↑ WUE (drought)	=Yield; ↑ acidity	Costa, 2022; Copp et al., 2025 [15,42]
Touriga Nacional/Franca	Mediterranean (Portugal)	3; 3 sprays	Pre-veraison	↓	↑ PSII (+67%); ↑ ETR	↑ Anthocyanins; ↑ acidity; ↓ pH	Dinis et al., 2016; Singh et al., 2020 [23,32]
Merlot	Semi-arid (USA)	3; 1 spray	Pre-veraison	↓	↑ WUE	↑ Berry weight; variable anthocyanins	Shellie & Glenn, 2008; Song et al., 2012 [20,25]
Viognier	Mediterranean (USA)	3; 1 spray	Pre-veraison	↓	↑ WUE (deficit)	↑ °Brix; ↑ alcohol	Shellie & Glenn, 2008 [25]
King Ruby seedless	Subtropical (Egypt)	2; 1–2 sprays	Pre-veraison	↓	↑ Defense response	↑ Berry firmness; ↑ quality	Rashad et al., 2023 [27]

Arrows represent the direction and magnitude of change (↑ increase, ↓ decrease).

The synthesis of flavonols is stimulated by UV radiation, and kaolin partially reflects this radiation. However, a decrease in these compounds was not observed; on the contrary, flavonol levels were maintained or even increased, suggesting that additional factors beyond UV radiation may influence their accumulation. This response may be due to kaolin reducing temperature, thereby enhancing enzymatic activity, leading to flavonol accumulation [40,43]. Although flavanols and flavonols increased in berries (skin, pulp, and seeds), these differences were not found in Mara wines [38].

Conversely, in Malbec grown under arid conditions, kaolin did not alter berry or leaf temperature, likely due to the extreme environmental conditions. However, the concentration of anthocyanins and total phenols increased [40], suggesting that kaolin may exert additional physiological effects beyond temperature modulation.

Furthermore, the aromatic profile of the grapes was also modified. As the temperature decreased, water and heat stress reduced and ripening occurred more slowly and evenly, thereby preserving varietal aromatic compounds [28,35,36,44].

In particular, terpenes and norisoprenoids tend to be better preserved under lower-temperature conditions due to reduced thermal degradation and oxidation. For instance, in cultivars such as Viognier and Sauvignon Blanc, kaolin application has been associated with improved aromatic balance and enhanced expression of varietal character [28,44].

Moreover, kaolin stimulated metabolic pathways, contributing to the biosynthesis of aromatic precursors [31,38,43,45]. These effects may contribute to an increased pool of aroma precursors in the berry, which can later be released during fermentation. However, the specific impact of kaolin on different classes of volatile compounds (e.g., esters, higher alcohols, or C_13_-norisoprenoids) remains insufficiently characterized and appears to be strongly dependent on cultivar and environmental conditions.

## 4. Phytosanitary Applications and Ecological Side Effects

In recent years, the reduction in the number of authorized active substances for pest control in agriculture has forced farmers to seek alternative strategies [46,47]. In this context, kaolin presents several advantageous properties, including its ability to reflect solar radiation and thereby impart a whitish appearance to the crop, hydrophobic nature, foliar adhesion that increases treatment persistence, and chemical inertness, which make it a valuable complementary tool within integrated pest management (IPM).

Consequently, kaolin is being used, often in combination with other substances, for controlling serious pests such as Olive fruit fly (*Bactrocera oleae*) [48], European pear psylla (*Cacopsylla pyri* (L.)) [49], Pistachio psyllid (*Agonoscena pistaciae*) [50], etc. Regarding its effectiveness compared with conventional insecticides, Nuñez-Lopez et al. [51] showed similar levels of Whitefly (*Trialeurodes vaporariorum*) in bean crops treated with kaolin and in those treated with systemic insecticides such as imidacloprid or diafenthiuron, suggesting that kaolin may be used as a sole phytosanitary substance for the control of certain pests.

The use of kaolin for its phytosanitary properties in viticulture has been investigated from various perspectives. For instance, Wang et al. [52], under laboratory conditions, reported that kaolin protects leaves by forming a surface barrier, directly impairing *Plasmopara viticola* sporangia/zoospore release and inducing plant defense responses. In the field, Rashad et al. [27] reported a reduction of up to 80.1% in downy mildew severity with 3% kaolin under their experimental conditions, and similarly confirmed defense activation through enhanced expression of defense-related genes and antioxidant responses, supporting kaolin as a practical, non-synthetic option for disease management. In terms of insect pest control, Pease et al. [53] reported a positive effect of kaolin in reducing egg hatching and larval development of *Lobesia botrana*. Although kaolin can contribute to reducing both pest pressure and disease severity and may therefore represent an alternative management strategy, several other substances are commonly employed for this purpose, such as copper-based products in the case of downy mildew or pheromones for the control of *L. botrana.* However, the increasing pressure of emerging and secondary pest species in viticulture has renewed interest in kaolin as a phytosanitary tool in viticulture, particularly against grapevine leafhoppers such as *Jacobiasca lybica* and *Empoasca vitis.*

**Figure 3:**
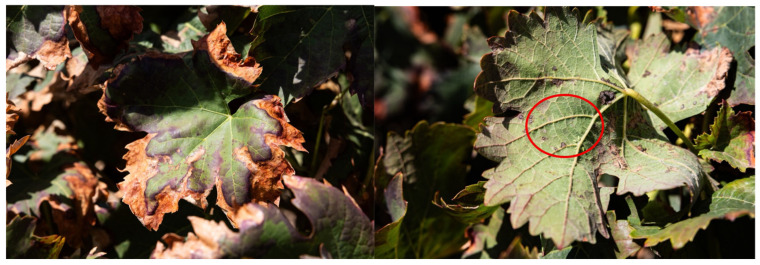
Characteristic leafhopper damage on grapevine leaves. Typical symptoms caused by leafhopper feeding, including marginal leaf necrosis (**left**). Nymphs at different developmental stages feeding on the abaxial leaf surface (**right**). The red circle shows the damage produced on the leaves.

Grapevine leafhoppers (Hemiptera: Cicadellidae) are piercing–sucking insects that feed on leaves using stylet mouthparts, primarily exploiting mesophyll cell contents, although ingestion from xylem and phloem tissues has also been reported [54]. Their feeding results in characteristic stippling and chlorotic spotting on the adaxial leaf surface and, under heavy infestations, may progress to leaf necrosis (“hopperburn”) and premature defoliation, compromising both yield and grape quality [55]. In addition to direct feeding injury, some leafhopper species may contribute to the epidemiology of grapevine phytoplasma diseases by acting as vectors [56]. Figure 3 illustrates typical leafhopper injury symptoms on the adaxial surface, together with four nymphs feeding on the abaxial side of the same leaf.

**Figure 4:**
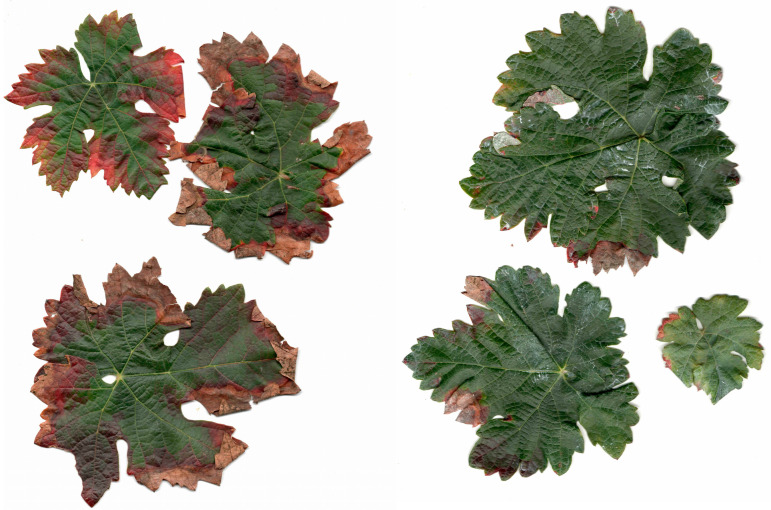
Untreated Termpranillo leaves (**left**), showing an average of 54% leaf area damaged by leafhoppers, compared with kaolin-treated leaves (**right**), which showed an average of 6% leaf area damage.

Kaolin particle films form a physical barrier on the leaf surface that can disrupt host acceptance and impair feeding activity, particularly in the nymphal stages of leafhoppers. In northern Italy, Prazaru et al. [57] demonstrated that kaolin was an effective tool for suppressing populations of the Nearctic leafhopper *Erasmoneura vulnerata* (Hemiptera: Cicadellidae) in organic vineyards. Similarly, Tacoli et al. [58] reported positive effects of kaolin applications on the control of *Empoasca vitis* and *Zygina rhamni* (Hemiptera: Cicadellidae). In a trial carried out on Tempranillo in Fontanars dels Alforins (Valencia, Spain) two applications of kaolin at 5% *w*/*v* clearly reduced leafhopper damage (Figure 4; unpublished data). In line with previous findings, the effectiveness of kaolin applications on grapevine crops was greater as a preventative measure in the second and third generations, compared with synthetic products [59,60]. This effect can persist up to 5 weeks if rainfall does not wash the product away [42].

Natural enemies shown to provide effective biological control include small egg-parasitic wasps (ca. 2 mm in wingspan) belonging to the family Mymaridae (Hymenoptera). Species of the genus *Anagrus* are among the most studied and are commonly associated with leafhopper pests of the genera *Empoasca* and *Erythroneura* [54]. Although no published studies have specifically reported parasitoids providing effective natural control of *Jacobiasca lybica*, available evidence suggests that generalist predators such as the green lacewing *Chrysoperla carnea* may play an important role in the biological control of this species [61]. Despite potential side effects of kaolin on natural enemies, Prazaru et al. [57] showed that kaolin applications did not negatively affect egg parasitoid activity (Mymaridae, including *Anagrus* spp.) or predatory mites (*Phytoseiidae*), which may contribute to overall biological control in vineyards. Similarly, Cargnus et al. [62] confirmed that kaolin treatments did not significantly affect predatory mites or the green lacewing *Chrysoperla carnea*. Moreover, Tacoli et al. [52] also reported that kaolin applications did not influence *Anagrus* spp. activity or egg parasitism rates under vineyard conditions.

While kaolin offers clear advantages and appears to be broadly compatible with biological control, its large-scale adoption in agriculture should be accompanied by a thorough evaluation of possible unintended effects. Because kaolin is an aluminum silicate mineral, its repeated use may contribute to aluminum accumulation in vineyard soils and leave mineral residues on grapes, which could influence aluminum levels in derived products (must and wine), although it is also used as an authorized clarification agent in wines, according to the OIV International Oenological CODEX. Linder et al. [38] evaluated this risk after foliar kaolin applications used to control *Drosophila suzukii* and found that aluminum concentrations in wine increased with kaolin dose; however, the maximum value measured (0.21 mg·L^−1^ after three applications at 2%) remained considerably below the OIV reference threshold of 8 mg·L^−1^. Nevertheless, data on aluminum accumulation under higher kaolin application rates, its potential presence in grape juice or must, and possible effects on the winemaking process beyond final wine composition remain scarce and warrant further investigation. Furthermore, it would be beneficial to determine if there is a potential long-term accumulation and the associated uncertainties.

Considering the relatively low impact of kaolin on natural enemies and its positive effects on vine physiological performance and overall grape and wine quality, kaolin appears to be a valuable tool for integration into IPM strategies in viticulture, particularly in vineyards where grapes are intended for winemaking.

## 5. Future Prospects and Challenges

While kaolin application has shown promising effects on grapevine physiology, fruit composition, and pest control, the current literature remains largely descriptive and, in many cases, lacks a mechanistic understanding of the processes involved. Furthermore, the variability of responses among cultivars, climatic conditions, and application strategies underscores the need for a more integrative, hypothesis-driven research framework. In this context, several key knowledge gaps can be identified, particularly regarding aluminum dynamics, the physiological mechanisms underlying the responses, the interaction between dose, genotype, and environment, and the potential effects on vineyard microbiomes.

Kaolin application, in particular, is receiving increasing attention due to its influence on the final quality of the wine, especially concerning the phenolic and aromatic composition of the grapes. Several studies have shown that, under certain environmental conditions, kaolin reduces heat stress, improves water use efficiency, delays ripening, increases acidity, and lowers pH. These effects, taken together, stimulate phenol biosynthesis and enhance aromatic potential. In red varieties, this effect is particularly relevant, as it allows wines to maintain their color intensity and antioxidant potential even in vintages marked by high temperatures during ripening. Furthermore, an improvement in aromatic potential has been observed, associated with greater stability of volatile precursors, which helps preserve varietal typicity in the context of global warming.

From the perspective of food safety and consumer perception, kaolin offers an additional advantage over other management strategies. As it is an inert and non-systemic material, the levels of residues detected in grapes, musts, and wines are extremely low, comparable to or even lower than those observed in untreated plots.

Additionally, the use of kaolin can indirectly contribute to greater stability in the viticultural agroecosystem by promoting the conservation of beneficial insects and reducing the selective pressure associated with the repeated use of synthetic pesticides. Its lack of toxicity to beneficial insects and soil microorganisms, along with its deterrent effect on certain pests, allows kaolin to be integrated into integrated pest management programs, reducing the risk of resistance and improving the resilience of the production system [54,57,58,61].

Despite these promising outcomes, a more critical analysis of the current literature reveals several important knowledge gaps that need to be addressed in order to establish kaolin as a reliable viticultural tool. One of the most relevant yet insufficiently explored aspects is the fate of aluminium derived from repeated kaolin applications. Although previous studies indicate that aluminium concentrations in wine remain below international regulatory thresholds [38], little is known about its chemical speciation in must and its potential impact on fermentation processes. Metal ions are known to influence yeast metabolism, enzymatic activity, and fermentation kinetics, suggesting that even low concentrations may have subtle effects on wine composition and stability [63,64,65]. Future research should therefore address aluminium partitioning between skins, must, and lees, as well as its interaction with microbial populations during alcoholic and malolactic fermentation. Another important limitation of current research lies in the lack of mechanistic evidence supporting several physiological responses attributed to kaolin application. Increases in Rubisco activity, improvements in photosystem II efficiency, and stimulation of secondary metabolism are frequently reported, yet the underlying processes are rarely quantified. In many cases, causal relationships are inferred from temperature reduction alone, without considering key physiological components such as mesophyll conductance, non-photochemical quenching, or changes in leaf energy balance [66,67]. A more comprehensive approach combining gas exchange measurements, chlorophyll fluorescence analysis, and biochemical profiling is required to disentangle these mechanisms and to establish robust cause–effect relationships. The interaction between kaolin dose, cultivar, and climatic conditions represents another major source of uncertainty. Although several studies acknowledge this variability, few have adopted experimental designs capable of disentangling these factors. Future research should prioritize multifactorial approaches, such as fully factorial field experiments combining different doses, genotypes, and environmental scenarios. In addition, modelling tools and response surface analyses could help identify optimal application strategies depending on specific objectives, such as maximizing anthocyanin accumulation, improving water use efficiency, or enhancing pest control efficacy [68,69]. Finally, the potential impact of kaolin on vineyard microbiomes remains virtually unexplored. As a persistent particle film applied to leaf and fruit surfaces, kaolin may alter the composition and activity of microbial communities in the phyllosphere, with possible consequences for plant health, disease dynamics, and grape quality. Moreover, indirect effects on soil microbiota through wash-off processes cannot be excluded. Given the increasing recognition of microbiomes as key drivers of vine performance and terroir expression, this represents a critical research gap that warrants dedicated investigation [70].

From an applied perspective, optimizing kaolin use in viticulture requires the integration of agronomic, physiological, and technological approaches. One of the main challenges is the definition of optimal application protocols considering dose, timing, cultivar, and climatic conditions. This could be addressed through the use of precision viticulture tools, including remote sensing and thermal imaging, to monitor canopy temperature and plant water status in real time.

In addition, biotechnological advances offer new opportunities to enhance kaolin efficiency. For instance, the combination of kaolin with other compounds such as silicon or biostimulants may improve plant stress tolerance through synergistic effects on physiological and hormonal regulation. Furthermore, advances in formulation technologies (e.g., particle size optimization, adhesion properties) could improve the persistence and effectiveness of kaolin films under field conditions. The integration of these approaches will be essential to develop tailored and efficient kaolin-based strategies for sustainable viticulture under climate change scenarios.

Taken together, the available evidence supports the use of kaolin as a strategic tool for adapting viticulture to future climate scenarios, not only for its mitigating effect on heat and water stress but also for its contribution to environmental sustainability, the quality of the final product, and the reduction in high-impact inputs. In this context, the development of application protocols tailored to specific soil, climate, and varietal conditions is emerging as a priority research area, with the aim of maximizing the benefits of kaolin and consolidating its use as a standard practice in the viticulture of the future.

## 6. Conclusions

Kaolin (KPF) is a sustainable and multifaceted tool for viticulture under current and future climate scenarios. By reflecting solar radiation, kaolin reduces leaf and berry temperatures, mitigates heat stress, and helps to preserve photosynthetic performance, especially in warm, water-scarce conditions. These physiological responses improve water use efficiency, although the magnitude of the effects depends largely on the climate, variety, and application strategy.

Kaolin application can affect grape composition and wine quality by delaying ripening under heat stress conditions, generally resulting in increased acidity and pH decreases. In cooler climates, it reduces sugar accumulation and potential alcohol levels. Simultaneously, kaolin has been shown to stimulate secondary metabolism, improving phenolic and anthocyanin content and promoting color stability and aromatic potential in wines produced at high temperatures. Studies conducted to date have associated low aluminum concentrations with the use of kaolin, even below regulatory thresholds. In addition to its physiological and oenological benefits, kaolin provides effective control of major vineyard pests, particularly leafhoppers, through a physical mechanism of action compatible with biological control and organic production systems.

Overall, kaolin represents an ecological and promising strategy for improving vine resilience and grape quality in the face of climate change. Future research focused on the interaction between dose, cultivar and climate effect will be essential to establish robust application guidelines and to consolidate kaolin use as a standard practice in sustainable viticulture.

## Data Availability

No new data were created or analyzed in this study.
